# Associations between Personal Attitudes towards COVID-19 and Public Space Soundscape Assessment: An Example from Antwerp, Belgium

**DOI:** 10.3390/ijerph182211774

**Published:** 2021-11-10

**Authors:** Francesco Aletta, Timothy Van Renterghem

**Affiliations:** 1 Institute for Environmental Design and Engineering, University College London, The Bartlett, 14 Upper Woburn Place, London WC1H 0NN, UK; 2 WAVES Research Group, Department of Information Technology, Ghent University, Technologiepark 126, B 9052 Gent, Belgium

**Keywords:** environmental noise, noise perception, soundscape, cycling, public space, survey, COVID-19

## Abstract

The COVID-19 pandemic, and the lockdown events and policies that followed, led to significant changes in the built environment and how it is experienced by people and communities. Among those, variations in the acoustic environments were some of the most noticeable in cities. This study investigated the relationships between the perception of the acoustic environment (i.e., soundscape) and different personal factors such as attitudes towards the pandemic and noise sensitivity, by performing a survey with 109 participants in an urban green public space in Antwerp (Belgium), shortly after most restrictions issued by the government were lifted in September 2020 when the first contamination wave ended. While preliminary in nature, the results of this data collection campaign show that people actively changing their behaviors (using less public transport or cycling more) assessed the soundscapes as less vibrant/exciting. People who were more concerned about the pandemic tended to notice more natural sounds and noise from traffic on nearby local roads. This same subset also put a bigger importance on the environmental quality of the public space than in the pre-pandemic period. Noise sensitivity also played a role, as an association was found between more-than-average noise sensitive persons and those more worried regarding the pandemic. Overall, the findings of this study confirm that at least part of the people have started to perceive the public space, including its soundscape, differently since the start of the pandemic.

## 1. Introduction

The COVID-19 pandemic events and all the consequent actions and policies implemented by governments and countries across continents have had (and to a great extent will still have in the foreseeable future) drastic impacts on how individuals, groups and communities relate to the built environment, and to environmental aspects more broadly [1]. There have been considerable changes in how people behave and interact, how social activities are performed and how the environment is experienced in cities, which have in turn multifaceted implications in the public health and societal dimensions Osborn, 2020) [2]. Early literature reviews of questions related to how people use public spaces in the context of the COVID-19 discourse seem to indicate that this pandemic will change public space design and use, and most importantly its perceptions, within and between cities [3]. Green areas in cities were of course a key public space people resorted to for leisure purposes during lockdowns (when allowed by local policy) as well as local commuting, as a preferred option to maintain better social distancing [4]. Some studies indeed focused on the role of green infrastructures as a public health “asset” during the pandemic. Hanzl observed that the pandemic stimulated academic and policy debate about future forms of urban living, and that temporary interventions and short-term adjustments introduced in response to the pandemic may actually indicate possible pathways for the future [5].

In particular, among all the environmental variations observed since the onset and development of the pandemic, sudden changes in noise levels (especially in urban contexts) were reported from different sources around the world (see for instance: [6,7,8,9,10,11]). In the environmental acoustics and soundscape research communities, this led to a growing interest to characterize changes in the sound environment of cities, both objectively (i.e., via acoustic measurements) and perceptually (i.e., via socio-acoustic surveys or alike) [12].

While noise level reductions due to the lockdown and social distancing policies imposed to counter the spread of the virus were generally acknowledged, what happened in terms of experience and perception of the sound environment as per the soundscape concept [13] was less clear. Maggi et al. [14] performed an online survey on the soundscape during the lockdown in Argentina and reported that, due to the decrease in mechanical sounds and increase in natural sounds, participants were more frequently conveying feelings of tranquility and happiness. Likewise, Bartalucci et al. [15] in another online survey conducted in Italy during different stages of COVID-19-related lockdowns reported an overall reduction in terms of noise annoyance that was likely induced by the limitation of traffic flows and human activities, which in turn led to improved soundscape assessments. On the other hand, studies focusing on the perception of acoustic environments for people forced to stay home occasionally highlighted negative outcomes, such as increased noise-induced psychological stress or increased rates of noise complaints (e.g., [16,17]).

Asensio et al. noted that lockdown soundscapes should probably be analyzed more from a sound source perspective, rather than a sound level-only perspective [18]. In perceptual terms, during the lockdown periods, people around the world generally reported natural sounds being heard more clearly because of less human-generated noise masking them. This seems to suggest some sort of decoupling between actual environmental sound levels and soundscape experiences by people, which could be due to collective psychological mechanisms triggered by pandemic-related events, as well as personal opinions and behaviors in response to policies imposed by governments. The effects that personal attitudes towards the COVID-19 itself, or the behavioral changes triggered by the policies aimed at containing the pandemic, had on how people relate with the environment is not fully understood. It is not clear whether such personal factors could modulate the way individuals experience and interpret environmental stimuli, of which soundscapes are a key component. Therefore, the aim of this study was exploring whether people with different attitudes towards the COVID-19 pandemic and/or different behaviors in response to the pandemic would assess the soundscape of a public space differently. For this purpose, a walking and cycling path bordering a highway in Antwerp (Belgium) was used as a case study and users were surveyed on different dimensions related to the perception of the sound environments, as well as personal stances towards COVID-19.

## 2. Materials and Methods

### 2.1. COVID-19 Situation at the Time of the Study

The first person who officially died in Belgium due to COVID-19 was announced by the Federal Government on 11 March 2020. Starting from 18 March on, a so-called “soft lockdown” was imposed by the Federal Government, including immediate shutdown of schools and pubs/restaurants, while only allowing food-related stores to stay open. In the Province of Antwerp, where the zone under study is located, the number of newly identified contaminations was at maximum 10 per 100,000 inhabitants per day during this first wave. Non-essential displacements by car were forbidden for the public at large, although walking/cycling outdoors was still allowed and even encouraged. During the month of May in 2020, these restrictions were lifted very gradually, and near the end of June, public life went more or less back to normal (with less than 1 contamination per 100,000 inhabitants per day).

Near the end of July 2020, local COVID-19 outbreaks in the Province of Antwerp (leading to a maximum of 12 new contaminations per 100,000 inhabitants per day), led to (moderate) local measures. At the beginning of September, these additional local restrictions were again lifted. At the time of the survey, near the end of September 2020, the sound pressure levels measured directly near the edge of the highway were almost equal to those in 2017 [19], which indicates that public life and economic activity was again at the same level as in the pre-pandemic period.

At the time of the survey, however, the number of contaminations were again exceeding 10 per 100,000 inhabitants per day in the Antwerp Province, and would lead to the peak of the second and more severe contamination wave to be reached early November 2020 (with 88 per 100,000 inhabitants per day). So, the survey was taken in between the first and second Belgian COVID-19 wave, after a local outbreak, and during a time window of a few weeks where these unprecedented restrictions on the public life were lifted to a large extent and daily life more or less normalized, but with the clear threat of a new contamination wave about to come.

### 2.2. Case Study

The cycling and walking path considered in this study is located in the Borgerhout district in the city of Antwerp (Belgium). It is (visually) immersed in vegetation and bordered by a depressed motorway (the Ring road) with a 6.3 m high embankment in between. The Ring road is by far the most noticeable source of environmental noise in the area, being a highly-trafficked multi-lane motorway, with a high share of heavy vehicles. The case study is interesting as it provides a unique context where the Ring road is a dominant source of environmental noise, on which traffic volumes were not affected by the lockdown policies at the time of the study. This is shown, e.g., by the measured difference in exposure level at the edge of the highway (behind the guardrail), relative to the pre-pandemic period, of only 0.1 dB (L_Aeq,1 month_) [19].

**Figure 1:**
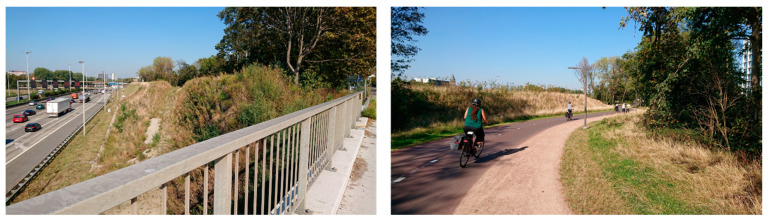
Photographs taken on site: a view towards the berm from the bridge (**left**), and another one along the path itself showing vegetation and the berm (**right**).

The part of the cycling/walking path under study has a length of approximately 560 m end to end, and it is comprised between two bridges (Luitenant Lippenslaan and Stenenbrug) that cross the Ring road. The area was recently the object of a landscaping renovation that made the Ring road invisible from the path, combined with a noise control intervention consisting of a raised vegetated berm (Figure 1), which was previously discussed in other studies [19,20]. This zone is characterized by strong presence of green features that play a moderating role in (negative) noise perception [21]. The landscaping/noise control intervention was completed at the time the COVID-19 pandemic events were developing, both worldwide and locally in Belgium, and the on-site survey was arranged shortly after in September 2020.

**Figure 2:**
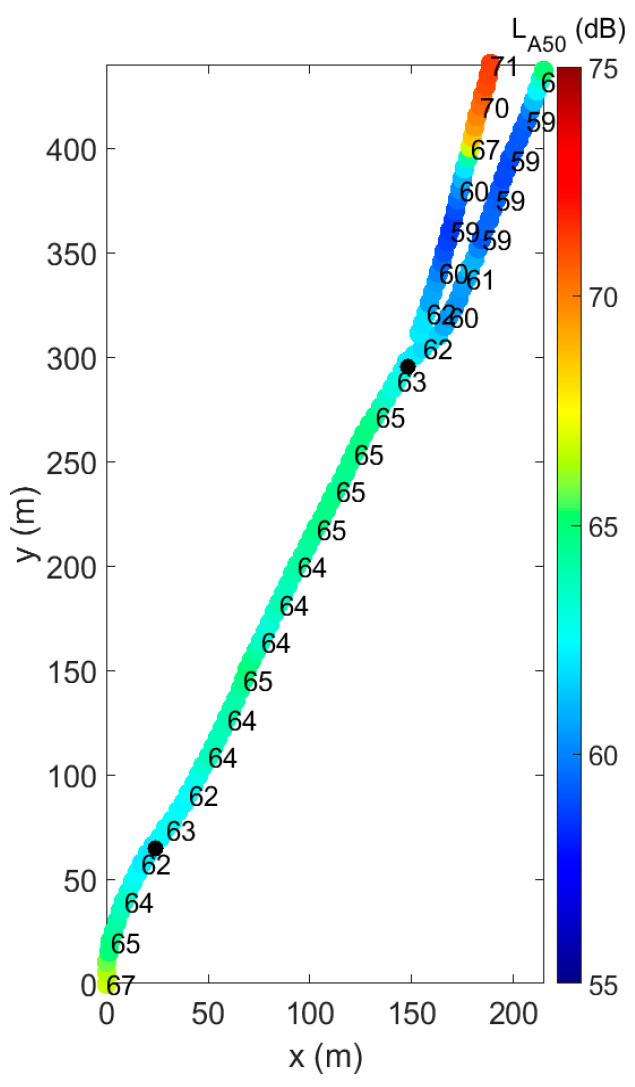
Spatial distribution of the sound pressure levels measured along the cycling path, using the L_A,50_ indicator during daytime hours in the week when the questionnaires were held. Distances are referenced to the bottom left position (x,y) = (0,0). The survey points are marked by the two black dots.

In Figure 2, detailed measurements of the sound exposure along the path are depicted, using a validated mobile measurement procedure as discussed in detail in Ref. [19]. Sound pressure levels, using the L_A,50_ indicator, range from 62 to 65 dBA in between the survey points.

### 2.3. Survey

**Figure 3:**
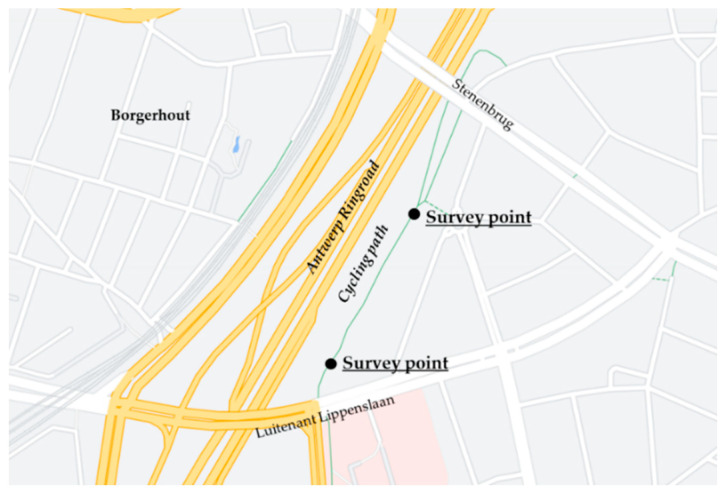
Plan of the case study area with survey points marked by black dots (map adapted from Google Maps); The length of the whole cycling/walking path is approximately 560 m.

On site surveys were carried out at the cycling path by two research students during the weekdays from 21 to 25 September 2020. Data collection sessions lasted approximately from 10:00 am to 06:00 pm. There were two survey points, one near the Lippenslaan bridge and another one at the path bifurcation towards the Stenenbrug end (Figure 3). The surveyors worked simultaneously and approached walkers/cyclists leaving the path according to their direction and position, to make sure responses to the questionnaire would reflect the environment as just experienced by the interviewee. Those who successfully completed the questionnaire were rewarded a small bicycle safety gadget as a token of appreciation for their time; completing the questionnaire usually took between 10 and 15 min. The selection of respondents was not targeted: surveyors on site would approach all passers-by if not already busy with another interview; the ratio of people deciding to participate was approximately one out of four of those approached. People were not approached if they were clearly wearing headphones on the assumption that they were not exposed to the urban sound environment under study.

The questionnaire comprised several sections that were proposed to participants who had agreed to contribute to the study after being approached by the research students and having provided informed consent. Several sections of the questionnaire were used for different studies, but in the context of this investigation, the relevant items (Table 1) that were considered are: (a) the overall experience of the place; (b) the soundscape appraisal; (c) noticed sounds; (d) the perceived loudness of the Ring road; (e) personal attitudes towards the COVID-19 and/or related behavioral changes; and (f) noise sensitivity. Items from categories (a), (b) and (c) are inspired by the ISO/TS 12913-2:2018 on soundscape data collection methods [22]. The perceived loudness question (d) was meant to assess the perceptual effects of the main environmental noise source on site (namely, the traffic noise coming from the near Ring road). The items of category (e) were drafted after internal discussion among the authors with the aim of using such variable to profile and/or stratify the sample of participants in different groups, namely: people who are “more concerned” rather than “less concerned” about the COVID-19 pandemic and its effects, also in relation to other environmental factors. Finally, the items of category (f) are based on a reduced Dutch version of Weinstein’s Noise Sensitivity Scale [20,23].

### 2.4. Data Analysis

The dataset used for this study consisted of the sample gathered via the questionnaires: 109 valid responses were provided by participants approached on-site. Cyclists represented 80% of the sample (87 out of 109 participants): preliminary comparisons between the cyclists and walkers’ groups revealed no statistically significant differences in perception so the two groups were pooled and treated together for the sake of analysis. The age of participants ranged between 18 and 81 years old (M = 47.0; SD = 14.8); gender: female = 45 (41%), male = 62 (57%), other/rather not say = 2 (2%).

For data analysis, the items of the questionnaire were treated as numerical or ordinal variables, except for the Yes–No questions in category (e) that were treated as dichotomous variables. The questions of category (e) were subjected to cluster analysis to help profiling the sample of participants (see Section 3.2). A two-step cluster analysis (SPSS IBM v.22) was performed on the scores of the four items in the category (e) of the questionnaire to define an “Attitude Towards COVID-19” (ATC19) variable. The clustering algorithm could handle either categorical or ordinal/numerical variables and considering the relatively small sample size, it was decided to force it into a two-cluster solution. The mean scores of the ATC19 items were inspected as a function of cluster membership; consequently, interpreting the four items of category (e), the two clusters were labelled as “More concerned about COVID-19” (1) and “Less concerned about COVID-19” (2), which were then in turn considered as categorical levels of the Attitude towards COVID-19 (ATC19) variable. Regarding the questions of category (f), a single number noise sensitivity score was obtained for each participant by averaging the answers to the 10 related questions. The all-sample average was 3.4 (1 = not sensitive at all; 5 = highly sensitive), thus a new categorical variable “Noise Sensitivity” was defined, and the sample stratified in two groups: “Low Sensitivity” (<3.5) and “High Sensitivity” (≥3.5); the scales of some items had to be flipped for coding purposes so that higher scores would always mean higher sensitivity. Stratifying noise sensitivity in two levels with this approach is considered reliable in previous studies (see, e.g., [24]).

## 3. Results

**Figure 4:**
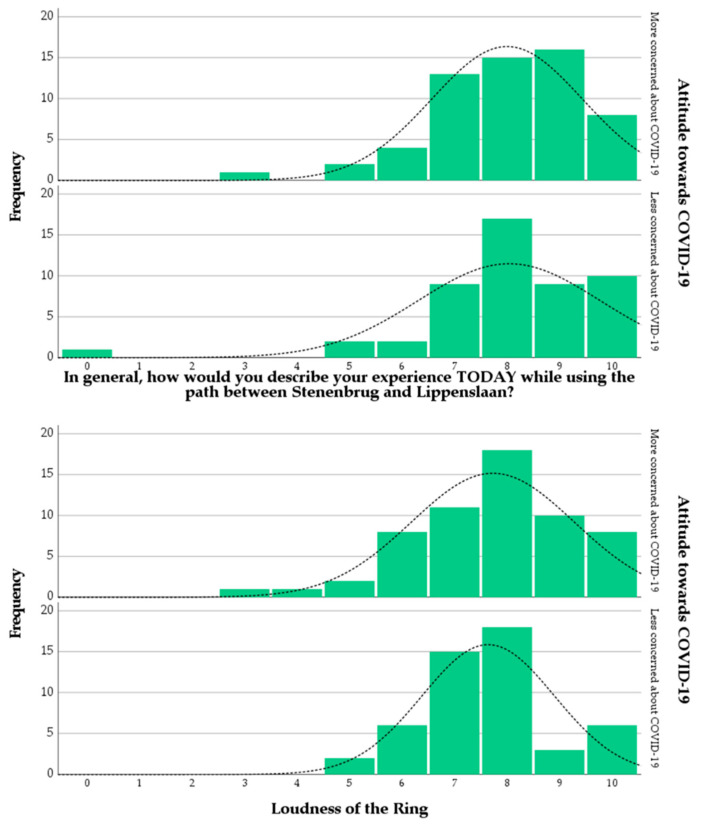
Distributions of the scores for the items of category (a) (**top**) and category (d) (**bottom**) of the questionnaire, split by ATC19.

**Figure 5:**
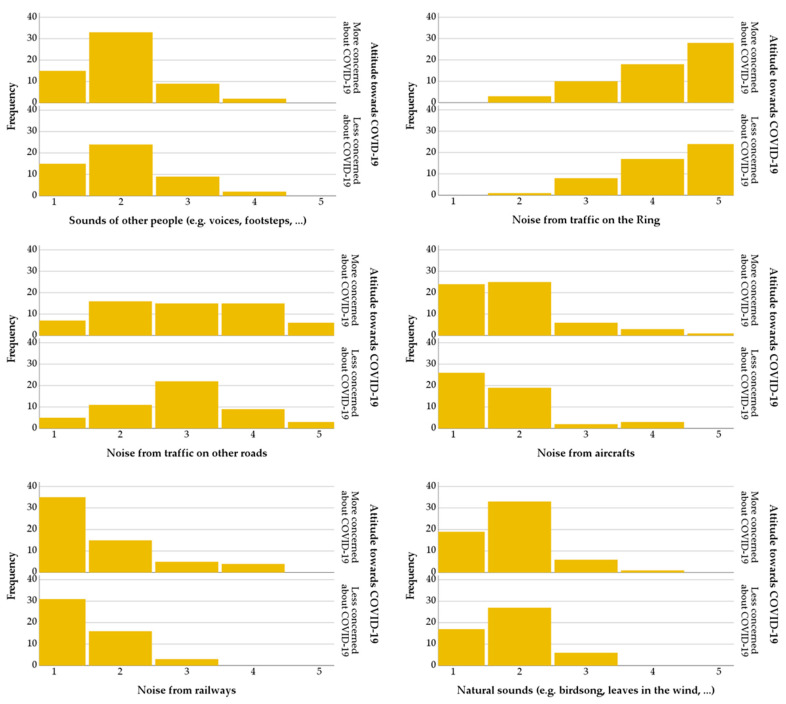
Distributions of the scores for the items of category (c) of the questionnaire (i.e., “Noticed sounds”), each split according to the ATC19 variable groups.

In terms of dataset description, Figure 4 shows the distributions of scores for the participants’ general experience of the place on the day of the survey, and the perceived loudness of the traffic noise coming from the Ring road, with the sample stratified by the ATC19 variable. The general experience of the respondents is relatively good for both the More concerned group (M = 8.00) and the Less concerned group (M = 8.04), even though the noise coming from the Ring road is perceived as loud (M = 7.73 and M = 7.64, accordingly). Figure 5 shows that for the perceived dominance of sound source types, for both ATC19 groups, the noise coming from the Ring road is certainly dominant, with traffic noise coming from other roads also being a contributor to the sound environment; sounds from people and nature were slightly noticeable, while noises from railways and aircrafts were mostly absent. The distributions of perceived dominance scores of sound sources look similar between ATC19 groups; a slightly different pattern can be observed for the “Noise from traffic on other roads” item, where for the “Less concerned about COVID-19” group, scores tend to accumulate around a middle score (3), while for the “More concerned about COVID-19” group, the sample seems to be more evenly distributed across the full 1–5 range of scores. This outcome is somehow difficult to interpret but it could signify people of the less concerned group having a tendency to return scores that are less extreme.

**Figure 6:**
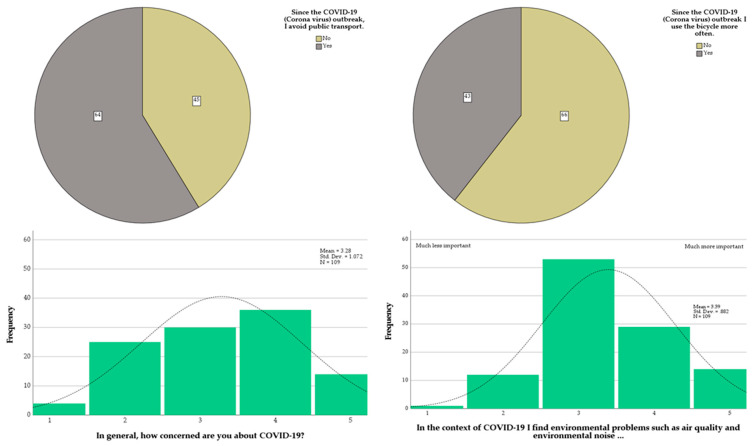
Distributions of the scores for the items of category (e) of the questionnaire.

In Figure 6, for the COVID-related questions in category (e), the sample was more or less evenly spread between people avoiding (or not) public transport since the COVID-19 outbreak, and people using more (or not) the bicycle. For the items about general concern about COVID-19 (i.e., third item of category (e)), as well as the relative concern regarding environmental issues (i.e., last item of category (e)), there was no clear consensus within the sample, with many occurrences around the neutrality scores.

### 3.1. Associations between Soundscape Scores and Attitudes towards COVID-19

In order to explore potential associations between experience/assessment of the sound environment and attitudes towards COVID-19 in the interviewed sample, a set of Spearman’s rank-order correlation tests were run to assess the relationships between the items in the questionnaire categories (b) and (c), and the items in the questionnaire category (e) (see Table 1). Preliminary analysis showed the relationships to be monotonic, as assessed by visual inspection of the scatterplots. It should be noted that for the Yes–No questions in category (e), running the Spearman correlation was seen as an application of a point-biserial correlation used to measure the strength and direction of the association between continuous variables (category (b)) and dichotomous variables (category (e)).

**Figure 7:**
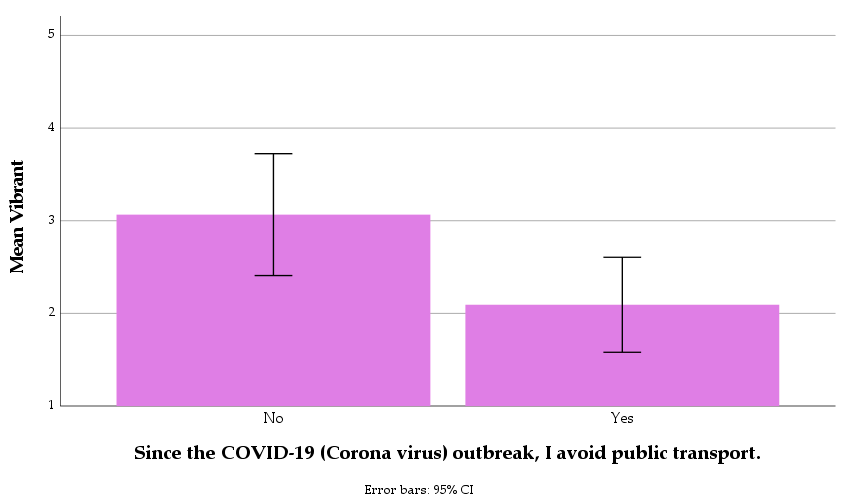
Mean scores for the soundscape attribute “Vibrant” between the two groups avoiding/not-avoiding public transport because of COVID-19.

**Figure 8:**
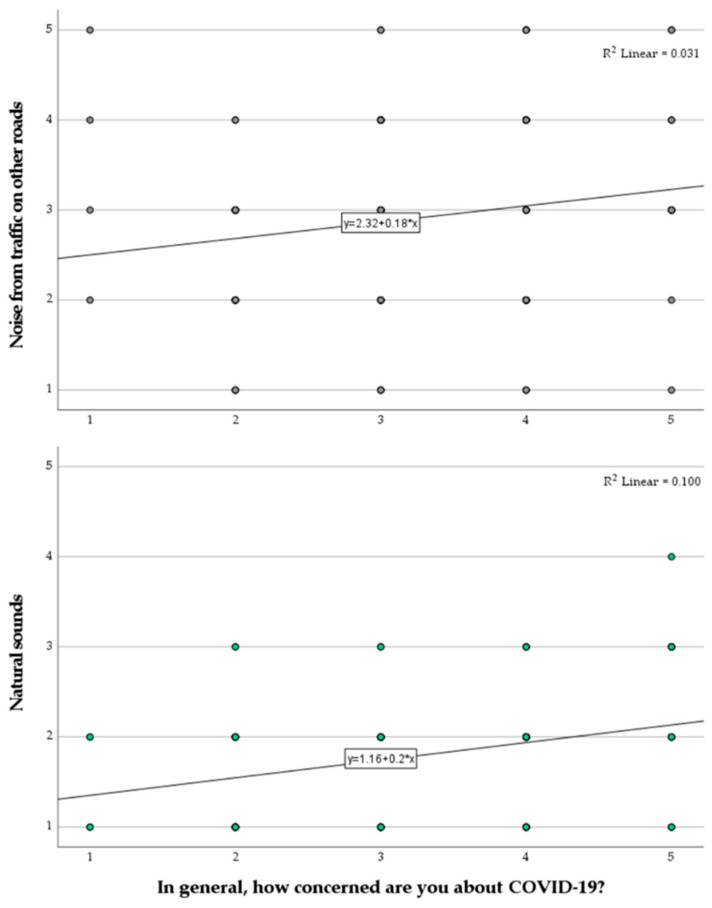
Perceived dominance of “Noise from traffic at other roads” (**top**) and “Natural sounds” (**bottom**), as a function of the scores for the item “In general, how concerned are you about COVID-19?” (1–5; Not at all–Very much).

Only three relationships resulted to be statistically significant, namely: a correlation between the “Since the COVID-19 (Corona virus) outbreak, I avoid public transport” item and the “Vibrant” item, *r_s_*(107) = −0.277, *p* = 0.018; a correlation between the “In general, how concerned are you about COVID-19” item and the “Traffic at local roads” item, *r_s_*(107) = 0.192, *p* = 0.045; and a correlation between the “In general, how concerned are you about COVID-19” item and the “Natural sounds” item, *r_s_*(107) = 0.300, *p* = 0.002. While other statistically significant correlations were observed within soundscape and COVID-19 categories (i.e., among items of the same category), no more of such relationships emerged across those. Furthermore, the lack of statistically significant correlation between the overall experience of the path (questionnaire category (a)) and the perceived loudness of the Ring road (questionnaire category (d)) led to the assumption that participants were able to respond on the questionnaire items in spite of the dominance of the traffic noise from the Ring road in the sound environment. The first of the three significant correlations described above is represented in Figure 7, where it can be seen that people avoiding public transport since the COVID-19 outbreak tended to score soundscapes as less vibrant. On the other hand, Figure 8 shows that with increasing concern about COVID-19, people tended to report noise from traffic at local roads and natural sounds as being more prominent, with a stronger effect for the latter sound source type.

The results of a logistic regression applied to the dataset show that participants who tend to avoid public transport are, as expected, much more concerned about COVID-19, i.e., returning a score of 3, 4, or 5 on the “In general, how concerned are you about COVID-19?” item (OR = 7.8; 95% CI = 2.9–20.7; χ^2^ = 19.5, *p* < 0.001). Logically, participants using the bicycle more since the pandemic are more likely to be concerned about COVID-19 (OR = 3.3; 95% CI = 1.2–9.0; χ^2^ = 5.8, *p* = 0.0158). More interestingly, those more concerned about COVID-19 state that environmental issues such as air quality and environmental noise (i.e., last item of category (e) of the questionnaire) have become more important (OR = 3.3; 95% CI = 1.2–9.0; χ^2^ = 5.8, *p* = 0.0158).

### 3.2. Associations between Attitude towards COVID-19 and Noise Sensitivity

**Figure 9:**
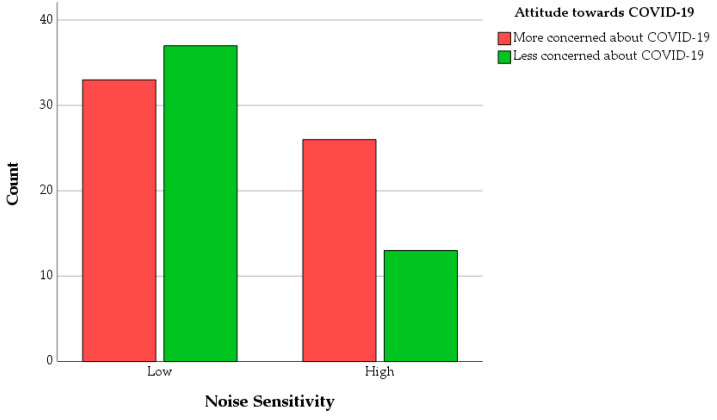
Bar charts of the number of Less and More concerned about COVID-19 participants (as per the ATC19 variable) as a function of Noise Sensitivity.

To gain further insights into the relationship between the experience of the pandemic and noise-related personal factors, a chi-square test for association was conducted between the ATC19 variable and the “Noise Sensitivity” variable. All expected cell frequencies were greater than five. There was a statistically significant association between the two variables, χ^2^(1) = 3.845, *p* = 0.050. This is reflected in Figure 9, where it can be observed that in the Low Noise Sensitivity group there are higher occurrences of participants less concerned about COVID-19, while there is an opposite trend, and with a bigger ratio, in the High Noise Sensitivity group, i.e., the sample of highly noise-sensitive participants mostly comprises people who are more concerned about the COVID-19 pandemic.

**Figure 10:**
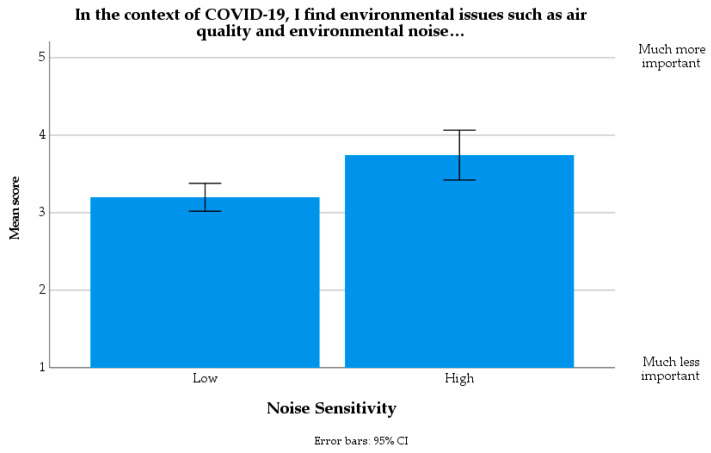
Mean scores for the item “In the context of COVID-19, I find environmental issues such as air quality and environmental noise…” (1–5; Much less important–Much more important), as a function of Noise sensitivity.

Since the level of significance in the chi-square test was marginal, a set of Spearman’s rank-order correlation tests were run between the Noise sensitivity variables and the single items of the ATC19 variable. The item that emerged to drive the significance outcome in the chi-square statistics was the one related to the general concern about COVID-19 in comparison with environmental pollution: the correlation between the “In the context of COVID-19, I find environmental issues such as air quality and environmental noise… (1–5; Much less important–Much more important)” item and Noise sensitivity resulted to be statistically significant, *r_s_*(107) = 0.307, *p* = 0.001. Figure 10 shows that the highly noise-sensitive group tended to consider environmental issues as more important since the pandemic, compared to the low noise sensitivity group.

A logistic regression model applied to the dataset revealed that the High Noise Sensitivity participants are more likely to be at least moderately concerned about COVID-19, i.e., returning a score of 3, 4, or 5 on the “In general, how concerned are you about COVID-19?” item (OR = 3.0; 95% CI = 1.2–7.3; χ^2^ = 6.14, *p* = 0.013).

## 4. Discussion

A survey was performed assessing the (acoustic) quality of a public space in the city of Antwerp (Belgium) in between two COVID-19 contamination waves. Although the public life was more or less normalized at the moment of the questionnaire, participants had been experiencing a disruption of society and their personal habits in an unprecedented way in the 5 to 6 months before. Note that walking or cycling outdoors was allowed at all times, and even encouraged by the Government, leading to the fact that many people rediscovered and re-appreciated the public space. Air quality and environmental noise, two environmental stressors with a direct impact on citizens, were considered to have become even more important overall in view of the pandemic, probably due to their increased use. These environmental issues do not seem to be downplayed in view of the more (direct) health threat the pandemic brought.

The hypothesis underlying this investigation was that, in the context of the collective experience of the pandemic and related policies, people would be on a relatively broad spectrum of mindsets related to COVID-19, and these could be to some extent reconnected to their experiences of outdoor environments in general, and the sound environments in particular, which were reported to have significantly changed in many news outlets around the world as the pandemic events unfolded. Recent research, for instance, showed that lockdown measures led to increased need for access to public green spaces close to the place of residence where people are seeking exposures to restorative soundscapes [25].

When looking at comparisons in terms of soundscape assessment and experience between groups with opposite attitudes towards the COVID-19 pandemic, the observed differences were actually limited, with individual responses deviating only on isolated perceptual items. The sound environment of the case study area was scored as less “Vibrant” by the group tending to avoid more the public transport because of COVID-19. This seems consistent with the current understanding of the vibrancy soundscape dimension, which is commonly associated with presence of people, music, and other human activities [26,27]. So, it looks sensible to assume that people avoiding public transport would somehow feel less socially connected and have fewer human interactions, leading to a reduced perceived vibrancy of their soundscapes.

The positive correlations between the level of general concern for the COVID-19 pandemic and increased perception of both natural sounds and noise from traffic on local roads could be seen as outcomes confirming that these people are more carefully listening to the environment, even in an acoustic environment dominated by traffic noise coming from the nearby Ring road. In other words, these people seem to be paying more attention, in a relative way, to their immediate surroundings and activities happening close to them.

The analysis of the association between noise sensitivity and attitude towards COVID-19 also revealed an interesting pattern that could be interpreted as people who self-report as more noise sensitive being also more generally concerned about environmental stressors and other external sources of stress/harm, such as the pandemic. This highlights that personal factors play an important role in the response to the sound environment, which is something that in soundscape studies has been extensively discussed also in relation to demographic traits and psychological state and well-being [28]. People who consider themselves as more-than-average sensitive to noise see this environmental stressor as being (even) more important since the unprecedented events of the COVID-19 pandemic. This also adds to the overall discourse around the noise sensitivity concept: can noise sensitivity be seen as a proxy for general sensitivity towards the environment? Previous studies had argued against this interpretation and concluded that noise sensitivity in general scarcely correlates with perception of the overall quality of the environments and would therefore only be related to the acoustic setting (see, e.g., [29]). However, Stansfeld and colleagues [30] recently showed that noise sensitivity is a specific predictor of psychological ill-health and could be part of a broader construct that they call “environmental susceptibility”. The current research seems to point in this same direction. This finding per se calls for more attention from environmental psychology and public health research, as well as a more integrated approach to the management and design of urban public soundscapes [31].

## 5. Conclusions

This study explored if people with different attitudes towards COVID-19 and different behaviors in response to the pandemic would assess the soundscape of a public space differently, and whether this could be correlated with a personal trait such as noise sensitivity. The overarching goal of this work is also starting a conversation around the interactions between soundscapes and more general changes caused by the pandemic happening at societal level. There is indeed growing international evidence that the pandemic affected the drivers that attract people to urban (green) public spaces [32] and how people relate to others in such contexts [33]. It is still unclear to what extent the pandemic permanently changed the way communities experience their soundscapes and to what degree they would go back to pre-pandemic conditions once this public health crisis is eventually overcome. In many countries, at the peak of lockdown restrictions, frequent calls were made to consider systemic change to urban mobility systems and general designs of urban spaces, but it is still early to determine what lessons urban planners and policy makers will extract from the pandemic [34]. At present, the main conclusions of this study are:

- Participants actively avoiding using the public transport since the outbreak of the COVID-19 tended to assess the soundscape of the investigated public space as less vibrant.
- Natural sounds and noise from traffic coming from local roads were assessed as being more present in the sound environment by participants who identified as being more concerned about COVID-19.
- Participants who were more concerned about COVID-19 reported environmental issues such as air quality and environmental noise to have become more important, suggesting that people may value the environmental quality of the public space more since the start of the pandemic.
- An association between people more sensitive to noise and more concerned about COVID-19 in general was observed.

The study has limitations, as the questionnaire used to stratify the sample on the COVID-related issues had to be extremely simplified due to time and logistic constraints of the on-site data collection campaign back in September 2020. It is also possible that the other participant-related factors, such as demographic or other personal traits would play a role in how people assessed the soundscapes in the context of the pandemic. However, while some information was available for the sample, it was decided not to investigate it due to its relatively small size, since such effects would likely require larger samples to be detected [28]. Establishing clear causal relationships with the available small dataset is of course difficult, as well as making conclusive inferences about the direction of the investigated relations (i.e., personal attitude affecting the soundscape, or vice versa); therefore, the findings in this study should be taken as a preliminary exploration of such associations. More studies with longitudinal data collection designs would be desirable to investigate them more comprehensively in the future. This will hopefully lead to a better understanding of these complex people-environment interactions.

## Figures and Tables

**Table 1:** Summary of the on-site questionnaire items used in this study. The order of categories does not reflect the order of presentation to participants, it is adapted for analysis purposes.

Category	Question(s)	Scale (Extremes)
(a) overall experience	How would you generally describe your experience today when using the path between Stenenbrug and Lippenslaan?	Very bad–Very good (0–10)
(b) soundscape appraisal	Overall, the acoustic environment you just experienced was… Eventful Vibrant Pleasant Calm Uneventful Monotonous Annoying Chaotic	Not at all–Completely (0–10)
(c) Noticed sounds	To what extent did you hear the following types of sounds: Other people (voices, footsteps, etc.) Highway traffic Traffic at local roads Airplanes Railways (trams and trains) Natural sounds (birds, wind, etc.)	Not at all–To a large extent (1–5)
(d) perceived loudness of the Ring road	When cycling/walking along the path, I rate the loudness of the environmental noise from the Ring road as…	Very quiet–Extremely loud (0–10)
(e) attitude towards COVID-19	Since the COVID-19 outbreak I avoid public transport Since the COVID-19 outbreak I use the bycicle more often In general, how concerned are you about COVID-19? In the context of COVID-19, I find environmental issues such as air quality and environmental noise…	Yes–No (1–0) Yes–No (1–0) Not at all–Very much (1–5) Much less important–Much more important (1–5) than before the pandemic
(f) Noise sensitivity	Please, state to what extent you agree to each of the following statements... No one should complain when one listens to music for a while I wake up quickly because of noise I get bothered when my neighbours are noisy I get used to most noises without much trouble Sometimes noise makes me nervous Music that I usually love bothers me when I am trying to focus I find it difficult to relax in a noisy place It does not matter what’s happening around me, I can always concentrate well I get angry with people making noise preventing me to sleep or work I am sensitive to noise	Do not agree at all—Totally agree (1–5)

## Data Availability

The data presented in this study are available on request from the corresponding author. The data are not publicly available due to no prior agreement with the funder.

## References

[1] Brown A., Horton R. (2020). A planetary health perspective on COVID-19: A call for papers. Lancet.

[2] Aletta F., Osborn D. (2020). The COVID-19 global challenge and its implications for the Environment–what are we learning. UCL Open Environ..

[3] Honey-Rosés J., Anguelovski I., Chireh V.K., Daher C., van den Bosch C.K., Litt J.S., Mawani V., McCall M.K., Orellana A., Oscilowicz E. (2020). The impact of COVID-19 on public space: An early review of the emerging questions–design, perceptions and inequities. Cities Health.

[4] Xie J., Luo S., Furuya K., Sun D. (2020). Urban Parks as Green Buffers During the COVID-19 Pandemic. Sustainability.

[5] Hanzl M. (2020). Urban forms and green infrastructure–the implications for public health during the COVID-19 pandemic. Cities Health.

[6] Lecocq T., Hicks S.P., van Noten K., van Wijk K., Koelemeijer P., de Plaen R.S.M., Massin F., Hillers G., Anthony R.E., Apoloner M.-T. (2020). Global quieting of high-frequency seismic noise due to COVID-19 pandemic lockdown measures. Science.

[7] Aletta F., Oberman T., Mitchell A., Tong H., Kang J. (2020). Assessing the changing urban sound environment during the COVID-19 lockdown period using short-term acoustic measurements. Noise Mapp..

[8] Asdrubali F., Brambilla G. (2021). Noise Mapping Special Issue: The noise climate at the time of SARS-CoV-2 Virus/COVID-19 Disease. Noise Mapp..

[9] Asensio C., Pavón I., De Arcas G. (2020). Changes in noise levels in the city of Madrid during COVID-19 lockdown in 2020. J. Acoust. Soc. Am..

[10] Steele D., Guastavino C. (2021). Quieted City Sounds during the COVID-19 Pandemic in Montreal. Int. J. Environ. Res. Public Health.

[11] Vida Manzano J., Almagro Pastor J.A., Garcia Quesada R., Aletta F., Oberman T., Mitchell A., Kang J. (2021). The “sound of silence” in Granada during the COVID-19 lockdown. Noise Mapp..

[12] Lee H.P., Kumar S. (2021). Perspectives on the Sonic Environment and Noise Mitigations during the COVID-19 Pandemic Era. Acoustics.

[13] International Organization for Standardization (2014). ISO 12913-1:2014 Acoustics—Soundscape—Part. 1: Definition and Conceptual Framework.

[14] Maggi A.L., Muratore J., Gaetán S., Zalazar-Jaime M.F., Evin D., Pérez Villalobo J., Hinalaf M. (2021). Perception of the acoustic environment during COVID-19 lockdown in Argentina. J. Acoust. Soc. Am..

[15] Bartalucci C., Bellomini R., Luzzi S., Pulella P., Torelli G. (2021). A survey on the soundscape perception before and during the COVID-19 pandemic in Italy. Noise Mapp..

[16] Tong H., Aletta F., Mitchell A., Oberman T., Kang J. (2021). Increases in noise complaints during the COVID-19 lockdown in Spring 2020: A case study in Greater London, UK. Sci. Total Environ..

[17] Torresin S., Albatici R., Aletta F., Babich F., Oberman T., Stawinoga A.E., Kang J. (2021). Indoor soundscapes at home during the COVID-19 lockdown in London–Part I: Associations between the perception of the acoustic environment, occupants’ activity and well-being. Appl. Acoust..

[18] Asensio C., Aumond P., Can A., Gascó L., Lercher P., Wunderli J.-M., Lavandier C., de Arcas G., Ribeiro C., Muñoz P. (2020). A Taxonomy Proposal for the Assessment of the Changes in Soundscape Resulting from the COVID-19 Lockdown. Int. J. Environ. Res. Public Health.

[19] Van Renterghem T., Thomas P., Dekoninck L., Botteldooren D. (2022). Getting insight in the performance of noise interventions by mobile sound level measurements. Appl. Acoust..

[20] Van Renterghem T., Aletta F., Botteldooren D. (2021). Changes in the Soundscape of the Public Space Close to a Highway by a Noise Control Intervention. Sustainability.

[21] Van Renterghem T. (2019). Towards explaining the positive effect of vegetation on the perception of environmental noise. Urban For. Urban Green..

[22] International Organization for Standardization (2018). ISO/TS 12913-2:2018 Acoustics—Soundscape—Part. 2: Data Collection and Reporting Requirements.

[23] Weinstein N.D. (1980). Individual differences in critical tendencies and noise annoyance. J. Sound Vib..

[24] Ekehammar B., Dornic S. (1990). Weinstein’s Noise Sensitivity Scale: Reliability and construct validity. Percept. Mot. Ski..

[25] Noël C., Rodriguez-Loureiro L., Vanroelen C., Gadeyne S. (2021). Perceived Health Impact and Usage of Public Green Spaces in Brussels’ Metropolitan Area During the COVID-19 Epidemic. Front. Sustain. Cities.

[26] Steele D., Bild E., Tarlao C., Guastavino C. (2019). Soundtracking the Public Space: Outcomes of the Musikiosk Soundscape Intervention. Int. J. Environ. Res. Public Health.

[27] Sun K., de Coensel B., Filipan K., Aletta F., van Renterghem T., de Pessemier T., Joseph W., Botteldooren D. (2019). Classification of soundscapes of urban public open spaces. Landsc. Urban Plan..

[28] Erfanian M., Mitchell A., Aletta F., Kang J. (2021). Psychological well-being and demographic factors can mediate soundscape pleasantness and eventfulness: A large sample study. J. Environ. Psychol..

[29] Schreckenberg D., Griefahn B., Meis M. (2010). The associations between noise sensitivity, reported physical and mental health, perceived environmental quality, and noise annoyance. Noise Health.

[30] Stansfeld S., Clark C., Smuk M., Gallacher J., Babisch W. (2021). Road traffic noise, noise sensitivity, noise annoyance, psychological and physical health and mortality. Environ. Health.

[31] Van Renterghem T., Dekoninck L., Botteldooren D. (2020). Multi-stage sound planning methodology for urban redevelopment. Sustain. Cities Soc..

[32] Ugolini F., Massetti L., Calaza-Martínez P., Cariñanos P., Dobbs C., Ostoić S.K., Marin A.M., Pearlmutter D., Saaroni H., Šaulienė I. (2020). Effects of the COVID-19 pandemic on the use and perceptions of urban green space: An international exploratory study. Urban For. Urban Green..

[33] Mehta V. (2020). The new proxemics: COVID-19, social distancing, and sociable space. J. Urban Des..

[34] Spennemann D.H., Parker M. (2020). Hitting the ‘pause’ button: What does COVID-19 tell us about the future of heritage sounds?. Noise Mapp..

